# Hydrogen activation using a novel tribenzyltin Lewis acid

**DOI:** 10.1098/rsta.2017.0008

**Published:** 2017-07-24

**Authors:** Robert T. Cooper, Joshua S. Sapsford, Roland C. Turnell-Ritson, Dong-Hun Hyon, Andrew J. P. White, Andrew E. Ashley

**Affiliations:** Department of Chemistry, Imperial College London, South Kensington Campus, London SW7 2AZ, UK

**Keywords:** frustrated Lewis pairs, hydrogenation, tin, Lewis acids, hydrogen activation

## Abstract

Over the last decade there has been an explosion in the reactivity and applications of frustrated Lewis pair (FLP) chemistry. Despite this, the Lewis acids (LAs) in these transformations are often boranes, with heavier *p*-block elements receiving surprisingly little attention. The novel LA Bn_3_SnOTf (**1**) has been synthesized from simple, inexpensive starting materials and has been spectroscopically and structurally characterized. Subtle modulation of the electronics at the tin centre has led to an increase in its Lewis acidity in comparison with previously reported R_3_SnOTf LAs, and has facilitated low temperature hydrogen activation and imine hydrogenation. Deactivation pathways of the R_3_Sn^+^ LA core have also been investigated.

This article is part of the themed issue ‘Frustrated Lewis pair chemistry’.

## 1. Introduction

Since the concept of ‘frustrated Lewis pairs’ (FLPs) was formalized a decade ago [1], there has been a rapid increase in interest and activity in this area of chemistry [2–4]. The combination of a Lewis acid and base (LA and LB, respectively), which are prevented from forming a strong classical adduct by steric and/or electronic factors [3], can possess unquenched reactivity that has been shown to allow activation of a range of small molecules. Following initial observations of the heterolytic cleavage of H_2_ [5–7] (which had traditionally only been achieved using transition metals), subsequent application to metal-free catalytic hydrogenation has brought great interest, with the scope progressing from imines, aziridines and protected nitriles substrates [8,9], to activated alkenes [10], and, most recently, aldehydes and ketones [11,12].

To date the vast majority of the focus in this diverse, rapidly expanding field of chemistry has been directed towards the use of boron-centred LAs [13]; by comparison equivalent FLP chemistry with heavy p-block elements has received far less attention [14–17]. Nevertheless, our current interest has been drawn to the use of stannylium ion R_3_Sn^+^ (R = alkyl) based LAs, which possess several properties very similar to the most commonly used LA in FLP chemistry, B(C_6_F_5_)_3_ [13]. In particular these LAs are isoelectronic, isolobal, and have been calculated to have comparable hydride ion affinities (Δ*G*_H−_ = 65.83 and 64.95 kcal mol^−1^ for ^n^Bu_3_Sn-H and [(C_6_F_5_)_3_B-H]^−^ respectively) [18], indicating that these species could show analogous reactivity in H_2_ activation and hydrogenation chemistry. Accordingly, we recently described the use of ^i^Pr_3_SnOTf (a surrogate for ^i^Pr_3_Sn^+^; OTf = CF_3_SO_3_) in FLP-mediated H_2_ activation chemistry when partnered with amine/pyridine LBs. Furthermore, ^i^Pr_3_SnOTf could successfully be employed in the catalytic hydrogenation of a variety of functional groups (C=C, C=N and C=O bonds), and demonstrated an unparalleled tolerance to moisture for FLP catalysis [14]. Nevertheless, we noted that one factor limiting the rate of some catalytic hydrogenations using ^i^Pr_3_SnOTf as a LA was the ease of H_2_ activation. In this respect, it is notable that earlier work by Manners and co-workers showed similar FLP systems based on ^n^Bu_3_SnOTf were unreactive towards H_2_, although they were capable of dehydrogenating amine-boranes. We proposed that this resulted from an overly strong interaction between the less bulky R_3_Sn^+^ and TfO^–^ moieties, which would result in significant quenching of the Lewis acidity at Sn [15]; this hypothesis drove us to pursue the synthesis of the more sterically encumbered yet electronically similar ^i^Pr analogue. These results clearly indicate that the reactivity of R_3_Sn^+^-based FLPs can be highly sensitive to the identity of R, and clearly there exists scope for further modification and optimization of such Sn(IV) LAs, which are appealing given their relative ease of synthesis, and the abundance and low cost of Sn [14].

Previously, computational calculations have reported that the successful activation of H_2_ is strongly correlated to the cumulative proton and hydride affinities of LB and LA, respectively [19], for which p*K*_a_ values of [LB-H]^+^ and Lewis acidity measurements (e.g. Gutmann–Beckett method) can be used as guiding experimental proxies. With this in mind, we speculated that increasing the Lewis acidity of R_3_Sn^+^ via the use of a more electron-withdrawing R group would facilitate faster FLP-mediated H_2_ activation and hence improved catalytic hydrogenation kinetics. Herein we report the targeted high yielding synthesis and characterization of the new LA Bn_3_SnOTf (Bn = PhCH_2_; **1**) which, by virtue of the greater inductive electron-withdrawing effect of the sp^2^-hybridized phenyl C atom versus an sp^3^-alkyl substituent, displays an enhanced Lewis acidity over ^i^Pr_3_SnOTf. Furthermore, we show that **1** displays facile H_2_ activation at lower temperatures than its ^i^Pr analogue under comparable conditions, and examine its activity for the catalytic hydrogenation of an imine substrate.

## 2. Experimental details

### (a) General considerations

Unless otherwise stated, all reactions were conducted under an inert atmosphere of dinitrogen using standard Schlenk techniques on a dual-vacuum-inlet gas manifold or MBraun DP Labmaster glovebox. All glassware was heated to 180°C overnight prior to use. All solvents were dried and degassed before use: pentane was dried using an Innovative Technology Pure Solv™ SPS-400 and stored over K; Et_2_O was distilled from Na/fluorenone and stored over K; CHCl_3_ was dried and stored over 3 Å molecular sieves; C_6_D_6_ and CDCl_3_/CD_2_Cl_2_ were freeze--pump--thaw degassed and dried over a K mirror and 3 Å molecular sieves, respectively. H_2_ was purchased from BOC (research grade) and dried by passage through a Matheson Tri-Gas Weldassure™ Purifier drying column. 2,4,6-Collidine (hereafter referred to as collidine) and Ph(H)C=NPh were purchased from major suppliers, degassed and dried over 4 Å molecular sieves before use. Bn_3_SnCl was purchased from Alfa Aesar and dried under vacuum. Benzyl chloride (BnCl), SnCl_4_, Mg, LiAlH_4_, I_2_ and trifluoromethanesulfonic acid (TfOH) were purchased from major suppliers and used as received.

### (b) Analytical measurements

NMR spectra were recorded on Bruker AV-400 MHz and DRX-400 spectrometers. ^1^H and ^13^C spectra were referenced internally to the residual solvent signals and reported in parts per million (ppm). ^19^F, ^31^P and ^119^Sn spectra were referenced externally to CFCl_3_, 85% H_3_PO_4(aq)_ and Me_4_Sn respectively. High resolution mass spectrometry was recorded using a Micromass Autospec Premier (EI mode) by Dr Lisa Haigh at Imperial College London. Single crystal X-ray diffraction data were collected and refined by Dr Andrew White (full details can be found in the electronic supplementary material). Elemental microanalysis was conducted by Stephen Boyer at London Metropolitan University.

### (c) Synthesis

#### (i) Tetrabenzylstannane (Bn_4_Sn)

A modified procedure of Smith & Kipping [20] and Huber and colleagues [21] was employed: SnCl_4_ (6.70 g, 25.71 mmol) was added slowly to Et_2_O (100 ml) at 0°C to give a milky-white suspension. Mg powder (2.50 g, 102.84 mmol) was added, followed by a single crystal of I_2_ (0.05 g, 0.20 mmol). Benzyl chloride (13.02 g, 102.84 mmol) in Et_2_O (80 ml) was added dropwise over a period of 90 min at 0°C. Following addition, the reaction was heated to reflux for 3 h followed by further stirring at room temperature for 24 h. The reaction was carefully quenched with water and the aqueous phase extracted with CHCl_3_. The remaining work-up was performed under air: the combined organic phases were dried over Na_2_SO_4_ and filtered, and the volatiles removed under reduced pressure resulting in an oil. Bn_4_Sn was crystallized from a slow cooled pentane solution at −45°C, affording 8.50 g (17.59 mmol) of a white crystalline solid in 68.4% yield.

^1^H NMR (400 MHz, CDCl_3_) *δ*: 2.22 [8H, s, ^2^*J*(^119,117^Sn-^1^H) = 58.3 Hz, CH_2_], 6.74 [8H, m, Ph], 7.01 [4H, m, Ph], 7.16 [8H, m, Ph]. ^119^Sn{^1^H} NMR (149 Hz, CDCl_3_) *δ*: −37.1 (s); these values are consistent with those previously reported [22].

#### (ii) Tribenzyltin triflate (Bn_3_SnOTf), (**1**)

Trifluoromethanesulfonic acid (TfOH, 0.63 g, 4.21 mmol) was added dropwise to a solution of Bn_4_Sn (2.14 g, 4.43 mmol) in CHCl_3_ (50 ml), causing the mixture to immediately become turbid. The reaction was stirred at room temperature for 18 h before the solvent was removed *in vacuo* and the solid subjected to a dynamic vacuum for 6 h. The solid was subsequently washed with pentane (4 × 15 ml) to furnish pure Bn_3_SnOTf as a white solid (2.01 g, 3.71 mmol) in 88% yield.

^1^H NMR (400 MHz, CDCl_3_) *δ*: 2.92 [6H, s, ^2^*J*(^117,119^Sn-^1^H) = 66.02 Hz, CH_2_], 6.79 [6H, m, Ph], 7.12 [3H, m, Ph], 7.20 [6H, m, Ph]. ^13^C{^1^H} NMR (101 MHz, CDCl_3_) *δ*: 26.8 [s, ^1^*J*(^117^Sn-^1^H) = 258.4 Hz, ^1^*J*(^119^Sn-^1^H) = 271.2 Hz, CH_2_], 118.9 [q, ^1^*J*(^19^F-^13^C) = 317.2 Hz, CF_3_], 125.9 [s, ^5^*J*(^117,119^Sn-^13^C) = 21.6 Hz, Ph], 128.1 [s, ^3^*J*(^117,119^Sn-^13^C) = 33.4 Hz, Ph], 129.4 [s, ^4^*J*(^117,119^Sn-^13^C) = 18.2 Hz, Ph], 135.9 [s, Ph]. ^19^F NMR (376 MHz, CDCl_3_) *δ*: −77.0. ^119^Sn{^1^H} NMR (149 Hz, CDCl_3_) *δ*: 87.4 [br s, Δν½ = 48.4 Hz]. Elemental analysis found (calculated) for C_22_H_21_O_3_F_3_SSn: C 48.69 (48.83), H 4.01 (3.91). HRMS (EI): *m/z* found (calculated) for C_22_H_21_O_3_F_3_SSn: 542.0202 (542.0186).

#### (iii) Tribenzyltin hydride (Bn_3_SnH), (**2**)

A modified procedure of Miura and colleagues [23] was employed for the independent synthesis of Bn_3_SnH (**2**): Bn_3_SnCl (1.00 g, 2.34 mmol) was added to LiAlH_4_ (0.08 g, 2.13 mmol) in Et_2_O (20 ml) at 0°C and stirred for 30 min. The suspension was filtered via cannula before the volatiles were removed *in vacuo*. The solid was extracted into pentane (3 × 10 ml) and filtered. The volatiles were removed under reduced pressure to furnish 0.294 g (0.75 mmol) of **2**, as a viscous oil in 37% yield, which solidified upon cooling to −20°C in a glovebox freezer for storage.

^1^H NMR (400 MHz, C_6_D_6_) *δ*: 2.17 [6H, d, *J* = 1.5 Hz, ^2^*J*(^117,119^Sn-^1^H) = 61.5 Hz, CH_2_], 5.71 [1H, sept, *J* = 1.5 Hz, ^1^*J*(^117^Sn-^1^H) = 1693.8 Hz, ^1^*J*(^119^Sn-^1^H) = 1773.1 Hz], 6.79 [6H, m, Ph], 6.94 [3H, m, Ph], 7.09 [6H, m, Ph]. ^13^C{^1^H} NMR (101 MHz, C_6_D_6_) *δ*: 17.9 [s, ^1^*J*(^117^Sn-^1^H) = 277.0 Hz, ^1^*J*(^119^Sn-^1^H) = 289.5 Hz, CH_2_], 124.2 [s, ^5^*J*(^117,119^Sn-^13^C) = 16.1 Hz, Ph], 127.9 [s, Ph], 128.9 [s, ^4^*J*(^117,119^Sn-^13^C) = 13.6 Hz, Ph], 142.1 [s, ^2^*J*(^117,119^Sn-^13^C) = 39.6 Hz, Ph]. ^119^Sn{^1^H} NMR (149 Hz, C_6_D_6_) *δ*: −85.4 (s); these values are consistent with those previously reported [23].

### (d) Gutmann–Beckett Lewis acidity measurements [24]

Et_3_PO (3.6 mg, 0.02 mmol) and Bn_3_SnOTf (32.4 mg, 0.06 mmol) were dissolved in CD_2_Cl_2_ (0.4 ml) and added to a NMR tube with a capillary insert containing 1 M Et_3_PO in CD_2_Cl_2_. Based on the ^31^P{^1^H} chemical shift of the resulting Et_3_PO adduct relative to the insert, the acceptor number (AN) was calculated using the formula of Mayer *et al.* [25] and Beckett *et al.* [26] AN = [(*δ*(sample) − 41.0] × [100/(86.14 − 41.0)]. ^31^P{^1^H} NMR, *δ*_adduct_ = 74.41 ppm gave an acceptor number of 74.0.

### (e) H_2_ activation procedure using Bn_3_SnOTf, (**1**) and collidine

Inside a glovebox **1** (16.2 mg, 0.03 mmol) and collidine (3.6 mg, 0.03 mmol) were combined in C_6_D_6_ (0.4 ml) and transferred into a NMR tube fitted with a Young's valve. The solution was freeze--pump--thaw degassed and H_2_ (1 bar) was admitted while the solution was at −196°C (which equates to a pressure of approximately 4 bar at room temperature) and the reaction was analysed by ^1^H, ^19^F and ^119^Sn spectroscopy. The reaction was then heated in an oil bath to 50°C for 2 h, after which it was reanalysed by NMR techniques. This revealed the formation of **2** by the diagnostic Sn-H septet resonance at *δ* = 5.71 ppm accompanied by ^117/119^Sn-^1^H satellites and the ^119^Sn resonance at *δ* = −85.4 ppm.

### (f) Investigations into PhCH_2_/H scrambling and deactivation routes

#### (i) Thermal stability of Bn_3_SnOTf, (**1**)

A sample of **1** (16.2 mg, 0.03 mmol) was dissolved in C_6_D_6_ (0.4 ml) and transferred into a NMR tube fitted with a Young's valve. The reaction was followed by ^1^H and ^119^Sn{^1^H} NMR spectroscopy but no reaction was observed, even after heating to 70**°**C for 72 h.

#### (ii) Thermal stability of Bn3SnOTf (**1**) and collidine

A NMR tube was loaded with **1** (16.2 mg, 0.03 mmol), collidine (3.6 mg, 0.03 mmol) and C_6_D_6_ (0.4 ml), which led to the formation of an adduct by ^119^Sn{^1^H} NMR spectroscopy. However, no further change was observed by ^1^H and ^119^Sn{^1^H} NMR spectroscopy, even after heating to 70**°**C for 72 h.

#### (iii) Thermal stability of Bn_3_SnOTf (**1**) and Bn_3_SnH (**2**)

Inside a glovebox **1** (16.2 mg, 0.03 mmol) and **2** (11.8 mg, 0.03 mmol) were combined in C_6_D_6_ (0.4 ml) and transferred into a NMR tube fitted with a Young's valve. The mixture was monitored by ^1^H and ^119^Sn{^1^H} NMR spectroscopy at regular intervals for 60 h at RT. Complete decomposition of **1** and **2** was observed with concomitant formation of Bn_4_Sn (^119^Sn{^1^H} NMR *δ* = −37.5 ppm), along with the formation of an intractable precipitate, after this time. An analogous reaction conducted with heating to 50**°**C for 5 h gave identical results.

#### (iv) Thermal stability of Bn_3_SnOTf (**1**), Bn_3_SnH (**2**) and collidine

**1** (8.1 mg, 0.015 mmol), **2** (5.9 mg, 0.015 mmol) and collidine (3.6 mg, 0.03 mmol) were combined in C_6_D_6_ (0.4 ml) and transferred into a NMR tube fitted with a Young's valve. The mixture was monitored by ^1^H and ^119^Sn{^1^H} NMR spectroscopy at regular intervals over the course of 60 h at RT, during which partial decomposition of **1** and **2** to Bn_4_Sn was observed (^119^Sn{^1^H} NMR *δ* = −37.5 ppm). An analogous reaction conducted with heating to 50**°**C for 5 h showed complete decomposition.

### (g) Imine hydrogenation procedure using Bn_3_SnOTf (**1**) and collidine

Inside a glovebox **1** (10.8 mg, 0.02 mmol), collidine (2.4 mg, 0.02 mmol) and Ph(H)C=NPh (**3**) (36.2 mg, 0.20 mmol) were dissolved in C_6_D_6_ (0.4 ml) and transferred into a Wilmad high pressure NMR tube fitted with a PV-ANV PTFE valve. H_2_ was admitted to a pressure of 10 bar (at room temperature) and analysed by ^1^H, ^19^F and ^119^Sn NMR spectroscopy. The reaction was heated in an oil bath to 50**°**C without active mixing and monitored at regular intervals. The conversion (%) was determined by relative integration of ^1^H resonances belonging to the amine product [PhC*H*_2_-NHPh, (**4**)], residual starting material [Ph(*H*)C=NPh, (**3**)]. This procedure was repeated at 70**°**C and room temperature.

## 3. Results and discussion

### (a) Synthesis and characterization of Bn_3_SnOTf **(1)**

**Figure 1.**
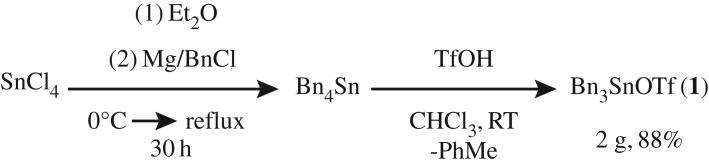
Synthesis of **1**.

**Figure 2.**
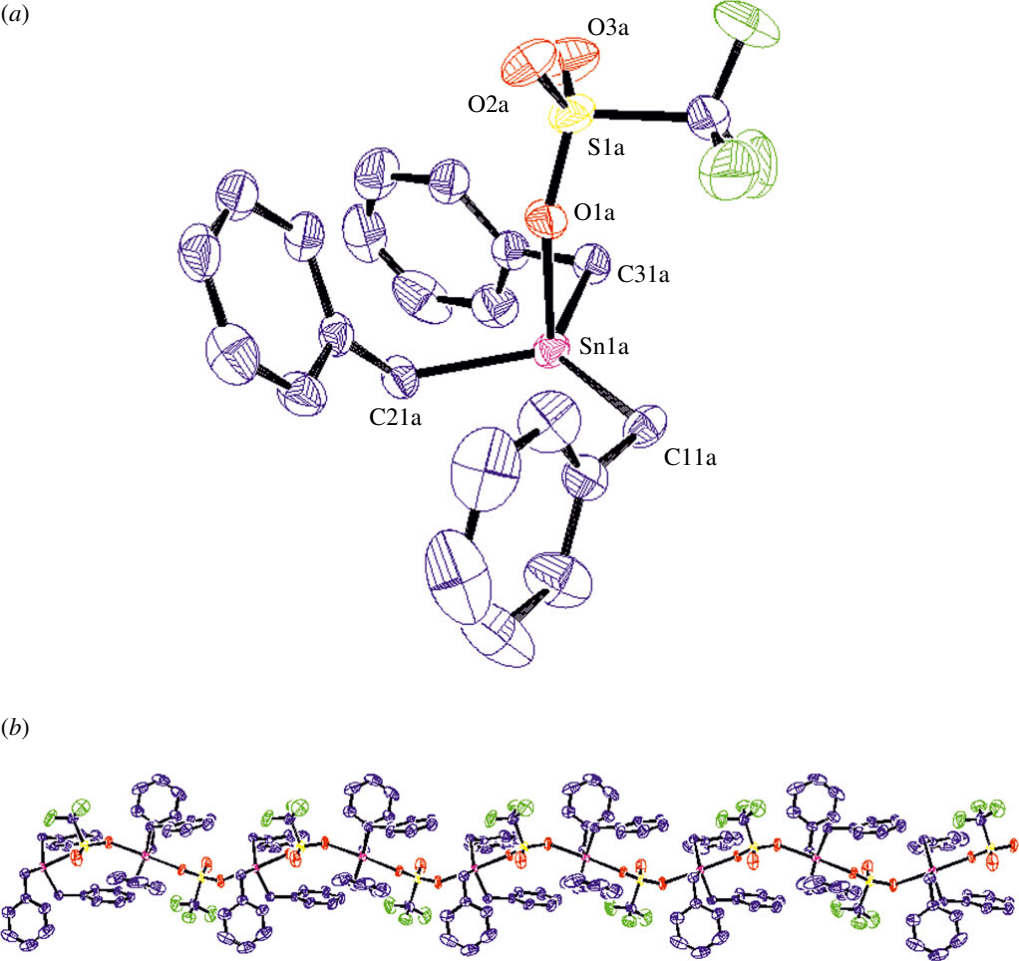
Molecular structure of 1 (molecule a). Ellipsoids shown at 50% probability, H atoms omitted for clarity (C atoms blue, O atoms red, F atoms green, S atoms yellow, Sn atoms pink). (*a*) View of one independent Bn_3_SnOTf fragment in the asymmetric unit. (*b*) Extended view of the polymeric structure generated from the independent fragment along the b axis (all four independent molecules form similar polymeric chains).

The target compound Bn_3_SnOTf (**1**) was synthesized by the facile proteodealkylation of Bn_4_Sn (synthesized by a modified procedure of Smith & Kipping [20] and Huber *et al.* [21]) with TfOH (figure 1). Subsequent work-up yielded **1** as a white solid in excellent yield (88%). **1** has been characterized by ^1^H, ^13^C, ^19^F and ^119^Sn NMR spectroscopy, elemental analysis, HRMS (EI) and X-ray crystallography. Single crystals were grown from a cooled (–20°C) saturated Et_2_O solution under an inert atmosphere, for which X-ray diffraction data were collected and refined, and the structure is shown in figure 2.

**1** crystallizes in the chiral space group P2_1_ and contains four independent molecules (molecules **1a**–**d**; see table 1 for more details) in the asymmetric unit, which are geometrically closely related. Each independent molecule forms its own unique extended polymer structure along the *b* axis, in which the TfO moieties bridge two separate Bn_3_Sn centres. The ligands are coordinated in a distorted trigonal bipyramidal arrangement around Sn, with the three benzyl groups occupying the equatorial positions and oxygens from the bridging triflate moieties occupying the axial positions. The degree of distortion from an idealized trigonal bipyramidal structure can be quantified using the parameter *τ* [28]; the range 0.94–0.96 obtained for the four independent molecules in **1** indicates a near perfect trigonal bipyramidal geometry (idealized *τ* = 1, versus *τ* = 0 for square-based-pyramidal).

**Table 1.** Selected bond lengths and angles for isomorphic **1** and Ph_3_SnOTf [27]. ESDs are given in parentheses.

	**1a**	**1b**	**1c**	**1d**	Ph_3_SnOTf
Sn–R length (Å)	2.141(6)	2.141(7)	2.147(7)	2.141(8)	2.104(6)
	2.147(7)	2.151(6)	2.157(7)	2.143(7)	2.117(6)
	2.154(7)	2.152(7)	2.164(6)	2.146(7)	2.118(5)
Sn–O length (Å)	2.310(4)	2.288(5)	2.317(5)	2.306(6)	2.310(4)
	2.345(5)	2.351(5)	2.352(5)	2.337(5)	2.375(4)
O–Sn–O angle (°)	177.7(2)	177.66(18)	176.83(19)	176.1(2)	175.6(1)
R–Sn–R angle (°)	113.4(3)	115.2(3)	114.4(3)	114.4(3)	116.2(2)
	118.0(3)	117.7(3)	117.0(3)	115.9(3)	117.4(2)
	128.3(3)	126.4(3)	128.3(3)	129.5(3)	126.3(2)

**1** is isostructural with Ph_3_SnOTf [27], one of only two triorganotin triflates that have previously been structurally characterized, the other being the molecular species [(Me_3_Si)_2_CH]_3_SnOTf [29]. These structural variations are likely attributed to the differing steric bulk of the R groups around Sn, with the very large [(Me_3_Si)_2_CH] substituents favouring a distorted tetrahedral geometry over a polymer which necessitates higher coordination numbers. For Ph and Bn, the substituents are small enough for the LA Sn centres to bond in a hypervalent manner, resulting in the polymeric 5-coordinate geometry. The Sn-C and Sn-O bond lengths within **1** and Ph_3_SnOTf are almost identical within experimental error [27]. However, the latter are considerably longer than those observed in [(Me_3_Si)_2_CH]_3_SnOTf (2.139(4) Å) [29], presumably because the lower coordination number enables closer approach of the triflate moiety, compared to the more sterically congested 5-coordinate species.

**1** is highly soluble in polar halogenated solvents, displays appreciable solubility in benzene, yet is completely insoluble in aliphatic hydrocarbon solvents. The solution-phase room temperature ^1^H NMR spectrum in the non-donor solvent CDCl_3_ reveals a notably downfield shift of the methylene resonance (*δ* = 2.92 ppm; CDCl_3_) compared to Bn_4_Sn (*δ* = 2.22 ppm; CDCl_3_), with the same trend observable for the methylene carbon resonances (^13^C NMR: *δ* = 26.8 and 18.9 ppm respectively), which reflects the enhanced electron deficiency upon substituting the benzyl for a weakly coordinating triflate ligand. This might be expected to result in significant stannylium ion character in **1**, which is usually typified by a strongly downfield ^119^Sn NMR chemical shift. However, the single broad resonance seen for **1** in the ^119^Sn{^1^H} NMR spectrum (*δ* = 87.4 ppm; Δν½ = 48.4 Hz) is considerably upfield relative to the value reported for [^n^Bu_3_Sn][CB_11_Me_12_] (*δ* = 454 ppm), which exhibits the least coordinated trialkylstannylium core reported to date [30], and the related trialkyltin triflates R_3_SnOTf (R = ^n^Bu [31], ^i^Pr [14]; *δ* = 168 and 156 ppm, respectively). These data, in combination with the ^1^*J*(^13^C-^119^Sn) values for the R_3_SnOTf compounds (R = ^n^Bu 383; ^i^Pr 316; Bn 258 Hz) where higher values are proposed to be an indicator of increasing stannylium character [31], might imply that **1** should be the weakest LA of the R_3_SnOTf series. However, the ^119^Sn NMR chemical shift is not a direct correlation to Lewis acidity and can be highly dependent on solvent, degree of aggregation in solution, and the substituents of the stannyl core [32]; in this instance it may be conceived that the propensity to aggregate within the solution-phase (in the absence of external strong donor species) is enhanced due to a more electron-deficient Sn core. A more rigorous, quantitative method was developed by Gutmann and Beckett which uses the change in ^31^P NMR chemical shift of Et_3_P=O upon coordination to a LA to provide an AN value, the magnitude of which positively correlates with Lewis acidity; this is a more reliable indicator of Lewis acidity since it is expected that the nucleophilic O atom in Et_3_P^+^–O^–^ is competent at displacing bridging OTf interactions, thus providing a valid assessment of the Lewis acidity of the Bn_3_SnOTf monomer. The AN value of **1** was experimentally determined to be 74.0, and indicates that Bn_3_SnOTf is significantly more Lewis acidic than the related R_3_SnOTf (R = ^n^Bu, ^i^Pr; AN = 64.2 and 68.0, respectively) [14], albeit less so than the commonly used FLP LA B(C_6_F_5_)_3_ (AN = 78.1). This indicates, as intended by design, that **1** is the strongest LA in the R_3_SnOTf series and a strong candidate for improved heterolytic H_2_ activation and subsequent reactivity.

### (b) Hydrogen activation studies of Bn_3_SnOTf (**1**)

**Figure 3.**
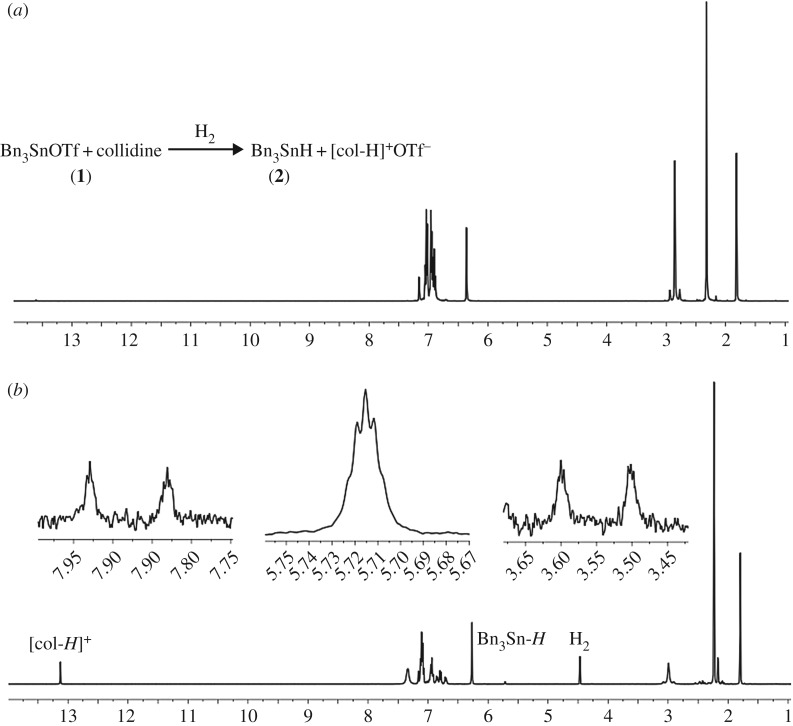
^1^H NMR spectra of **1** and collidine in C_6_D_6_ before (*a*) and after (*b*) admission of H_2_ (4 bar). Insets show Sn-*H* resonance and ^1^*J*(^117/119^Sn-^1^H) satellites.

Combination of **1** and collidine in a 1 : 1 ratio in C_6_D_6_ led to an upfield shift in the ^119^Sn{^1^H} resonance from *δ* = 74.9 to 33.6 ppm (br), concomitant with a slight shift of the ^1^H NMR resonances of **1**, consistent with a donor–acceptor interaction. Nevertheless, it is well known that certain ordinary Lewis pair adducts can exhibit FLP reactivity, as exemplified by the classical adduct between lutidine and B(C_6_F_5_)_3_ which generates the free FLP upon heating which subsequently cleaves H_2_ [33]; furthermore strongly bound adducts such as the silylium/phosphine species [^i^Pr_3_Si-P^t^Bu_3_]^+^ (for which no stable FLP counterpart can exist) can also engage in H_2_ heterolysis [34] (note that in our previous studies the ^i^Pr_3_SnOTf/DABCO (DABCO=1,4-diazabicyclo[2.2.2]octane) Lewis pair was found to activate H_2_ despite evidence for similar adduct formation [14]). With this in mind, admission of H_2_ (4 bar, 50°C, 2 h) led to the appearance of resonances in the ^1^H NMR [5.72 ppm, SnH, ^1^*J*(^117/119^Sn-^1^H) = 1693/1773 Hz; 13.13 ppm, NH] and ^119^Sn{^1^H} NMR (−84.8 ppm) spectra, which are consistent with formation of Bn_3_SnH (**2**) and [col-H]^+^[TfO]^−^, formed through H_2_ activation by the **1**/collidine Lewis pair (figure 3). These resonances were verified by comparison with literature values [23], and the independent synthesis of **2**. Significantly, this is only the second reported example of H_2_ activation using a Sn(IV) based LA.

It is interesting to note that the equivalent H_2_ activation by ^i^Pr_3_SnOTf and collidine required higher pressure (10 bar) and longer times (20 h) [14]. Similarly, no H_2_ activation was reported with ^n^Bu_3_SnOTf in combination with the stronger amine base 2,2,6,6-tetramethylpiperidine (TMP) [15], even with prolonged heating (18 h, 50°C), which is consistent with the suggestion from our Gutmann–Beckett measurements that **1** is the strongest LA in the R_3_SnOTf series (R = ^n^Bu, ^i^Pr, Bn).

Inspection of ^1^H NMR integrals revealed incomplete conversion of **1** to **2** (17% as ascertained by ^1^H NMR using a 2,5-dimethylfuran insert), which contrasts with the outcome of similar reactions using B(C_6_F_5_)_3_ whose H_2_ activation reactions have been reported to proceed to completion when using similarly strong LBs [19,33], along with a downfield shift and concomitant broadening of the C*H*_2_Ph resonances in **1**. However, similar observations were reported for ^i^Pr_3_SnOTf, which is nonetheless catalytically active for a variety of C=X (X = C, N, O) bond hydrogenations [14]. It was noted that alongside appreciable H_2_ activation at 50°C, several other new resonances were observed in the ^1^H and ^119^Sn{^1^H} NMR spectra (such peaks had not been observed during earlier investigations using ^i^Pr_3_SnOTf). These were postulated to result from deactivation/decomposition pathways operating within the system, which could provide one explanation for the incomplete conversion of **1** to **2**. As such, the origin of these resonances was investigated further.

### (c) Investigations into potential Bn_3_SnX (X = H, TfO) deactivation pathways

On closer inspection of the hydrogen cleavage experiments the diagnostic chemical shifts of Bn_4_Sn were noted in the ^1^H and ^119^Sn{^1^H} NMR spectra (*δ* = 2.17 and −37.5 ppm respectively; C_6_D_6_). This, in combination with the incomplete conversion of **1** to **2**, led us to conclude that additional side-reactions must also be operating. The thermal stability of **1** was established with and without the presence of collidine; samples remained stable (no change observed by ^1^H and ^119^Sn{^1^H} NMR) even at temperatures above those used for H_2_ activation (70 versus 50°C). Our initial suspicion related to decomposition of the hydride **2**; although **2** has been demonstrated to be an effective reagent in many organic transformations [23], it is known to be unstable at elevated temperatures [35], converting smoothly to hexabenzyldistannane (Bn_3_SnSnBn_3_) with concomitant evolution of H_2_ [36]. However, at no time was this distannane observed by NMR spectroscopy, indicating that **2** does not decompose via this pathway.

The mutual compatibility of **1** and **2** were subsequently probed (1 : 1, C_6_D_6_) which led to an instant interaction as evidenced by the broadening of both sets of resonances (most notably methylene) in the ^1^H NMR spectrum (electronic supplementary material, figure S6*a*). After 30 min at RT there were distinctive resonances indicating the formation of Bn_4_Sn and after a further hour, broad resonances at *δ* = 9.01 and 5.28 ppm appeared (albeit in low intensity), the latter being identical to that previously reported for Bn_2_SnH_2_ [37]. Interestingly, the diagnostic downfield resonance is almost identical to the related diorganotin hydride species, Bu_2_Sn(H)OTf (*δ* = 8.99 ppm; C_6_D_6_) [38], strongly indicating the possibility that a benzyl-substituted analogue might be transiently formed. The resonances for Bn_4_Sn steadily grew in intensity at room temperature, while those for **1** and **2** decreased, until after 60 h the dominant species present was Bn_4_Sn; when this reaction mixture was heated to 50°C from the start rapid conversion of **1** and **2** to Bn_4_Sn is seen; in both instances a solid material precipitated which proved to be intractable in all non-reacting solvents tested. The same conditions were then applied to a sample of **1** and **2** with collidine also present. After 30 min at RT no change was noted except the expected formation of the **1**-collidine adduct, as witnessed in §3(b); while Bn_4_Sn was similarly observed to appear and grow in intensity concomitant with the decrease of **1** and **2**, this was at an appreciably retarded rate in comparison with the results in the absence of collidine. After 60 h at RT the predominant species in the ^1^H and ^119^Sn{^1^H} NMR spectra were **1** and **2**; only after heating at 50°C for a further 5 h did Bn_4_Sn become the predominant species. Interestingly, the resonance at *δ* = 5.28 ppm attributed to Bn_2_SnH_2_ was again present, and a characteristic resonance attributed to [col-H^+^] had appeared in significant intensity (*δ* = 13.38 ppm), upon heating.

**Figure 4.**
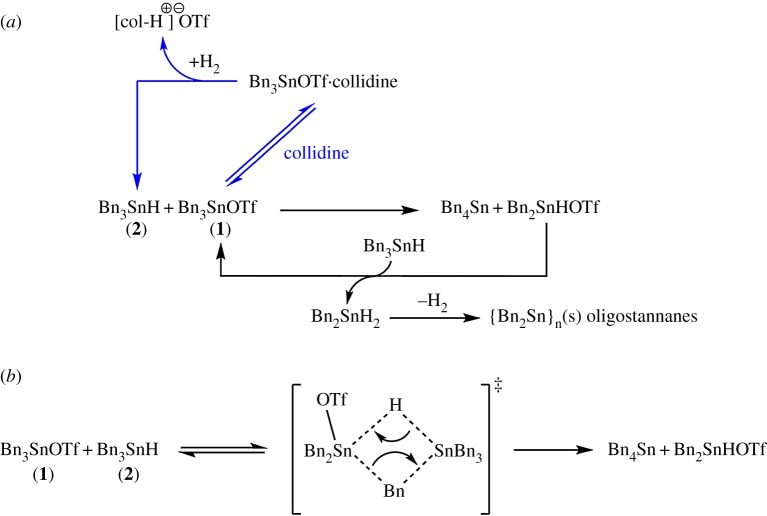
(*a*,*b*) Proposed deactivation pathways for Bn_3_SnX (X = TfO, H; **1**, **2**) during H_2_ heterolysis reactions.

Based on the observations above and the lack of any formation of Bn_3_SnSnBn_3_ or dibenzyl (Bn_2_), a proposed mechanism which rationalizes the incomplete **1**/collidine-mediated H_2_ heterolysis reaction, and observed decomposition products, is outlined in figure 4*a*.

It is postulated that the combination of **1** and **2** leads to formation of a binuclear complex (figure 4*b*), as evidenced by a broadening of resonances in the ^1^H NMR spectrum. Rearrangement of this intermediate through PhCH_2_/H exchange between Sn centres could then lead to formation of the observed Bn_4_Sn and Bn_2_Sn(H)OTf (corresponding to the *δ* = 9.01 ppm resonance in the ^1^H NMR spectrum). Bn_2_Sn(H)OTf is expected to be highly reactive by analogy with its ^n^Bu analogue (hence its low steady-state intensity). Subsequently Bn_2_Sn(H)OTf reacts with the more powerful hydride donor **2**, which reforms **1** and accordingly produces the observed Bn_2_SnH_2_ (^1^H NMR: *δ* = 5.28 ppm). R_2_SnH_2_, especially those containing R groups of a low steric threshold (e.g. ^n^Bu, Bn) are also highly reactive compounds which are prone to decomposition via a radical chain mechanism, forming H_2_ and complex mixtures of oligo/polystannanes {R_2_Sn}*_n_*, which for Ph-rich species can display poor solubility, especially when cross-branching occurs [from dehydrocoupling with (PhCH_2_)_3_SnH] [39]; the latter explains the observation of a precipitate and overall loss of ^1^H NMR signal intensity during the reaction, as the PhCH_2_ groups are sequestered from solution.

The stabilizing effect when collidine is present may thus be explained by its ability to form a **1**-collidine adduct which competes with, and effectively retards, the formation of the complex between **1** and **2**, thereby inhibiting the rate of decomposition; this result corroborates the idea that facile formation of the binuclear complex is key to the deactivation mechanism. It should also be emphasized that the presence of [col-H]^+^ in this reaction can be rationalized from the **1**/collidine-mediated cleavage of H_2_, the latter formed from decomposition of Bn_2_SnH_2_, which leads to **2** (which re-enters the cycle) and the build-up of [col-H^+^], as observed. It is hence plausible that this constant syphoning of **2** from the system via ligand scrambling and subsequent decomposition may explain the incomplete conversion of **1** to **2** during the normal H_2_ activation reaction by **1**/collidine under a H_2_ atmosphere (§3(b)). Finally, H_2_ activation by the incipiently formed Bn_4_Sn, in conjunction with collidine, can be discounted since an independent experiment of this combination under H_2_ failed to show any reaction; this observation is in line with the poor Lewis acidity of tetralkyltin compounds.

### (d) Imine hydrogenations

Despite the observation of subsequent decomposition, the successful activation of H_2_ encouraged us to proceed to investigate the use of **1** in FLP hydrogenation reactions. It was hoped that, although **2** may exist only transiently in the presence of a suitable unsaturated substrate, the rate of reduction might exceed the rate of any undesirable side-reactions. Thus, as an attempted proof-of-principle, initial attention was focused on the imine Ph(H)C=NPh, (**3**), which is an archetypal substrate for FLP-mediated catalytic hydrogenations. When H_2_ (10 bar) was added to a solution of imine **3** and **1**/collidine (10 mol%) in C_6_D_6_ and the solution subsequently heated to 50°C, hydrogenation was indeed observed, with formation of amine PhCH_2_NHPh (**4**) (13.9% conversion after 128 h; table 2, entry 2); it is of note that imine hydrogenation using ^i^Pr_3_SnOTf required significantly higher temperatures (120°C) for any reaction under analogous conditions [14].

**Table 2.** **1**-catalysed hydrogenation of Ph(H)C=NPh. (Online version in colour.) 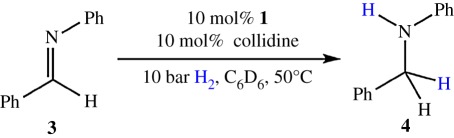

entry	temperature (°C)	time (h)	conversion (%)
1	25	128	4.3
2	50	128	13.9
3	70	49	13.2

Unfortunately, only low conversion is observed, with the rate of conversion decreasing as a function of time (5 h 6%; 21 h 10.1%; 66 h 13.3% and 128 h 13.9%), concomitant with the formation of resonances attributable to Bn_4_Sn, and hence with decomposition of **1**/**2** (vide supra). Nevertheless, this is still one of a very small number of examples of FLP-type hydrogenation using a p-block LA catalyst based on an element other than boron [14,40,41].

Attempts to increase the rate of reaction and percentage of imine conversion by raising the temperature to 70°C led to no more than minor improvements in rate (table 2), and resulted in the complete decomposition of **1** (via **1**/**2**) to Bn_4_Sn (as observed by ^1^H and ^119^Sn{^1^H} NMR spectroscopy). Conversely, at ambient temperatures decomposition was observed to a much lesser extent, but at the expense of even poorer conversion (4.3% after 128 h); nonetheless this result does demonstrate that H_2_ activation occurs even at room temperature for the **1**/collidine system. Ultimately, however, it seems clear that the potential for **1** to engage in useful FLP hydrogenation catalysis is unfortunately limited by the reduced chemical stability of the Bn_3_Sn core (relative to ^i^Pr_3_Sn, for example).

## 4. Conclusion

The Sn-based LA, Bn_3_SnOTf, has been synthesized in excellent yield and fully characterized in both the solution and solid state. X-ray crystallography reveals an extended polymeric structure with triflate anions bridging two Bn_3_Sn cores and a 5-coordinate, trigonal bipyramidal, geometry around the Sn centre; this is a rare example of a structurally characterized triorganotin triflate. Subtle modulation of the electronics at the Sn centre by incorporating benzyl instead of the more common alkyl ligands on the R_3_Sn core has allowed for a successful increase in R_3_SnOTf Lewis acidity, as evidenced by the comparison of AN values calculated from the Gutmann–Beckett spectroscopic method. This increase in Lewis acidity was further corroborated by the lower temperature and pressure at which Bn_3_SnOTf activates hydrogen in comparison with previously reported alkylated analogues. Competing decomposition/deactivation pathways preclude stable H_2_ activation for use in catalysis at elevated temperatures, however; these were probed and it was posited that a binuclear complex forming between Bn_3_SnOTf and the product of H_2_ activation, Bn_3_SnH, leads to ligand scrambling and ultimately deactivation to Bn_4_Sn. Future efforts are focused on the synthesis of new triorganotin LAs which display enhanced Lewis acidity to mediate rapid H_2_ activation, yet retain a high degree of thermal stability, for use as FLP hydrogenation catalysis.

## Supplementary Material

Supporting Information

## Data accessibility

Additional data supporting this article have been uploaded as part of the electronic supplementary material. Crystallography data of **1** has been deposited in the CCDC, no. 1534626. They are available online.
