# Supplementary material for: Hydrogen activation using a novel tribenzyltin Lewis acid
Source: Philos Trans A Math Phys Eng Sci. 2017 Jul 24;375(2101):20170008. doi: 10.1098/rsta.2017.0008 (PMC5540841; doi:10.1098/rsta.2017.0008)
Supplement: Supporting Information [file rsta20170008supp1.pdf]

Hydrogen Activation Using A Novel Tribenzyltin Lewis Acid

Supplementary material for R. T. Cooper, J. S. Sapsford, R. C. Turnell-Ritson, D.-H. Hyon, A. J. P. White, A. E. Ashley. 2017. Hydrogen Activation Using A Novel Tribenzyltin Lewis Acid. *Phil. Trans. R. Soc. A.* (doi: 10.1098/not yet assigned)

Contents **Page**

1. Characterisation of compounds S2
   1.1. NMR spectra of Bn_4Sn S2
   1.2. NMR spectra of Bn_3SnOTf (**1**) S3
   1.3. High resolution mass spectrum data of **1** S4
   1.4. NMR spectra of Bn_3SnH (**2**) S5
2. H_2 activation by **1** and collidine S6
3. Investigations into decomposition routes S8
   3.1. Thermal stability of **1** and **2** S8
   3.2. Thermal stability of **1**, **2** and collidine S11
4. Hydrogenation of imine **3** with **1** and collidine S13
5. X-ray crystal structure of **1** S15
6. References S18

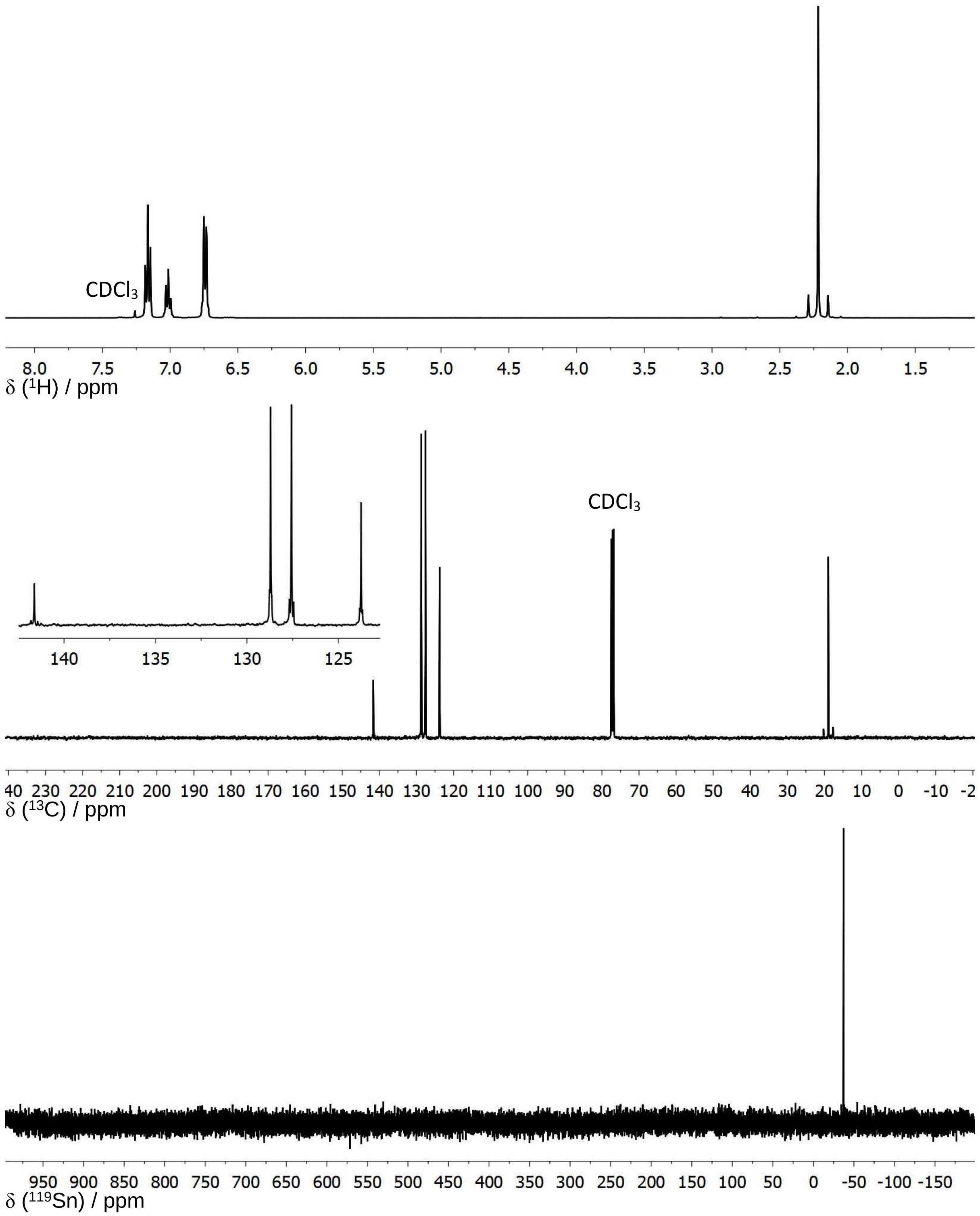

Supplementary Figure 1: ^{1}H, ^{13}C{^{1}H} and ^{119}Sn{^{1}H} NMR spectra of Bn_4Sn in $CDCl_3$.

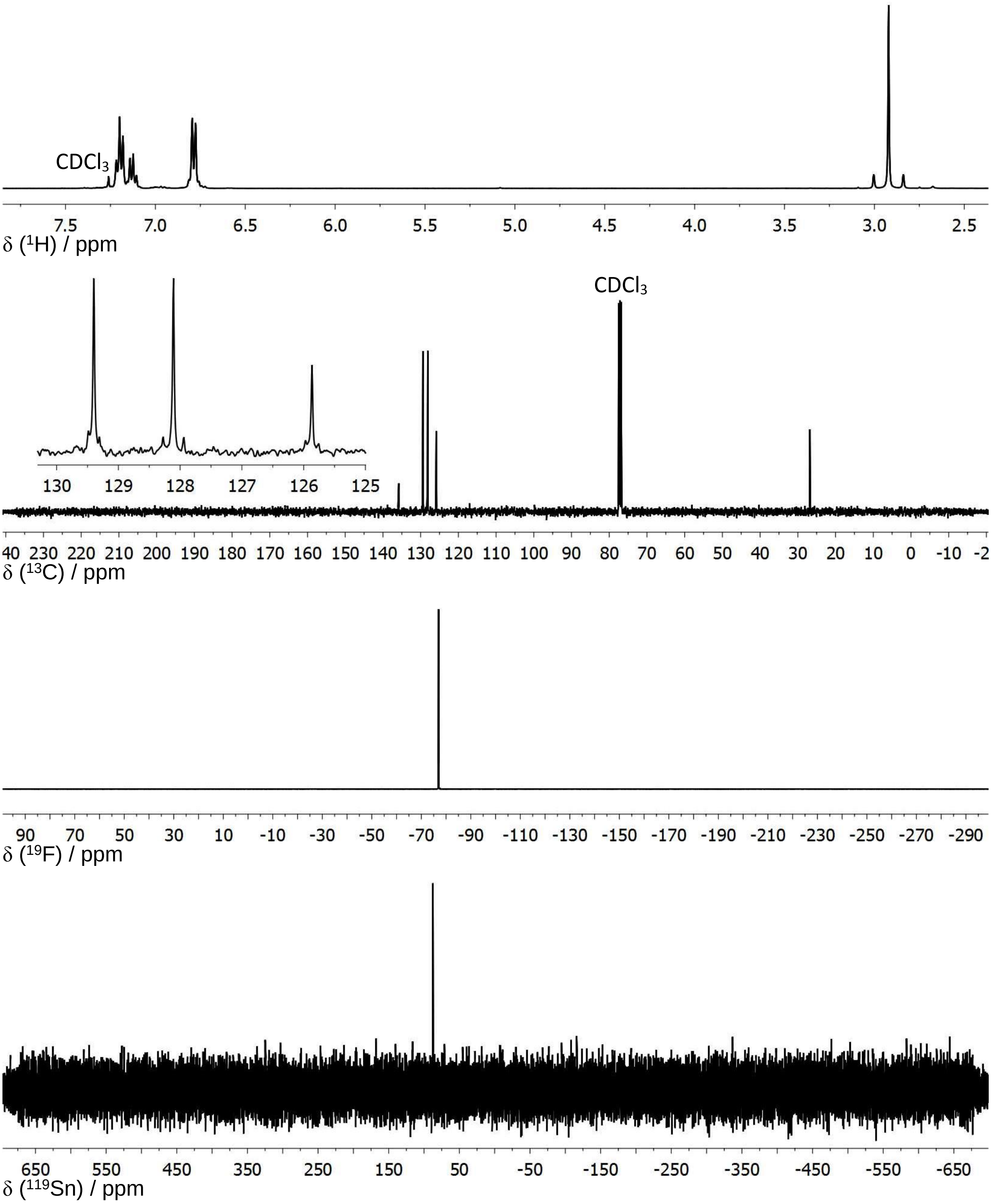

Supplementary Figure 2: ^{1}H, $^{13}C\{^{1}H\}$, ^{19}F and $^{119}Sn\{^{1}H\}$ NMR spectra of **1** in $CDCl_3$.

Elemental Composition Report **Page 1**

Single Mass Analysis (displaying only valid results)
Tolerance = 10.0 PPM / DBE: min = -1.5, max = 50.0
Selected filters: None

Monoisotopic Mass, Odd and Even Electron Ions
336 formula(e) evaluated with 1 results within limits (up to 30 closest results for each mass)
Elements Used:
C: 22-22 H: 0-150 N: 0-10 O: 0-10 S: 1-1 F: 3-3 120Sn: 0-2

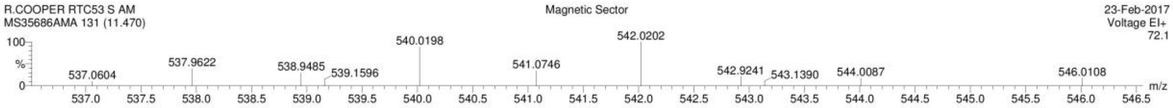

Minimum:				-1.5		
Maximum:		7.0	10.0	50.0		
Mass	Calc. Mass	mDa	PPM	DBE	i-FIT	Formula
542.0202	542.0186	1.6	3.0	12.0	1.8	C22 H21 O3 S F3 120Sn

Supplementary Figure 3: High resolution single mass analysis of **1**.

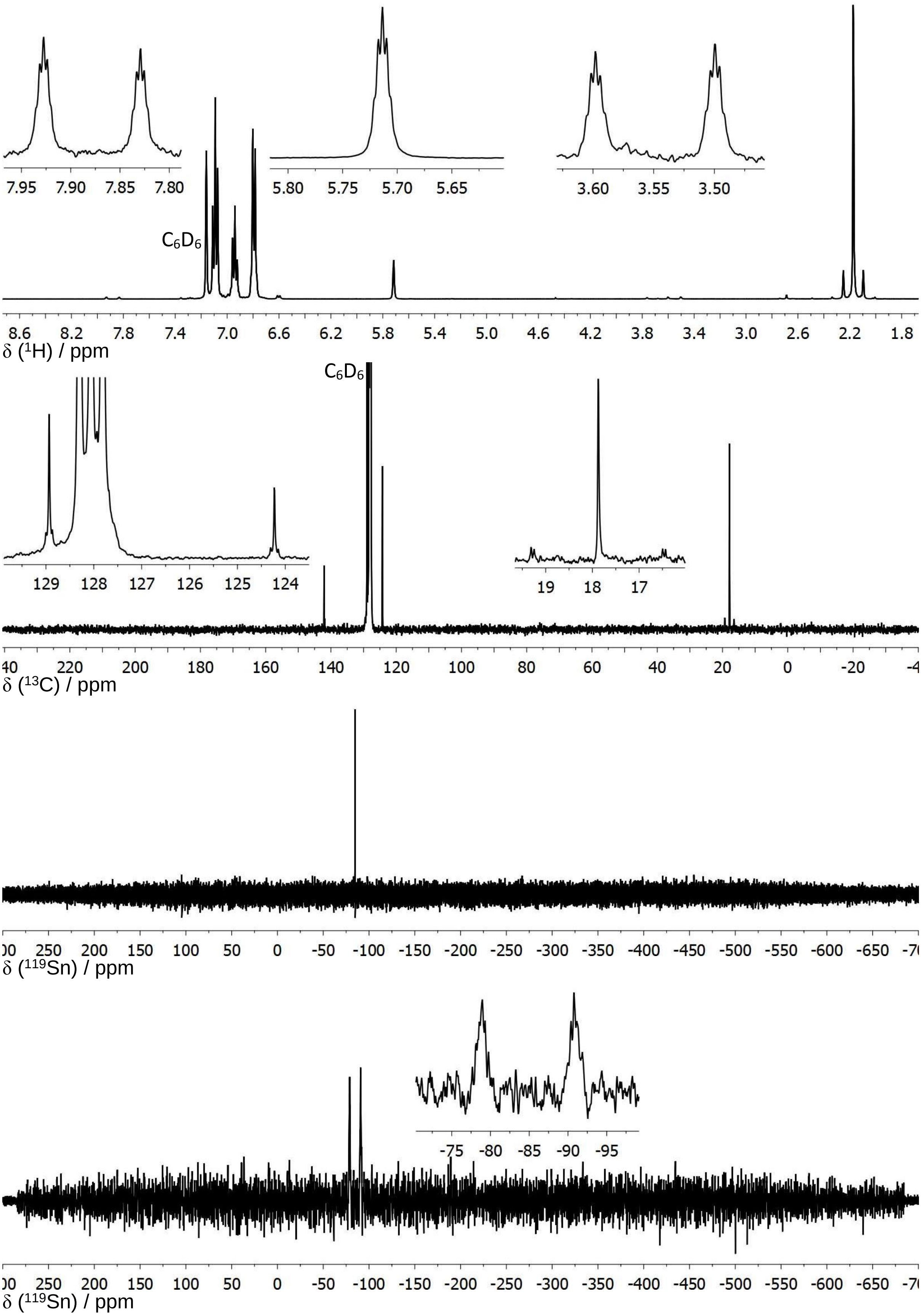

Supplementary Figure 4: ^{1}H [with insets showing the Bn_3Sn*H* multiplicity and $^{1}J(^{117/119}Sn-^{1}H)$ satellites], $^{13}C\{^{1}H\}$, $^{119}Sn\{^{1}H\}$ and ^{119}Sn NMR spectra of an authentic sample of **2** in C_6D_6.

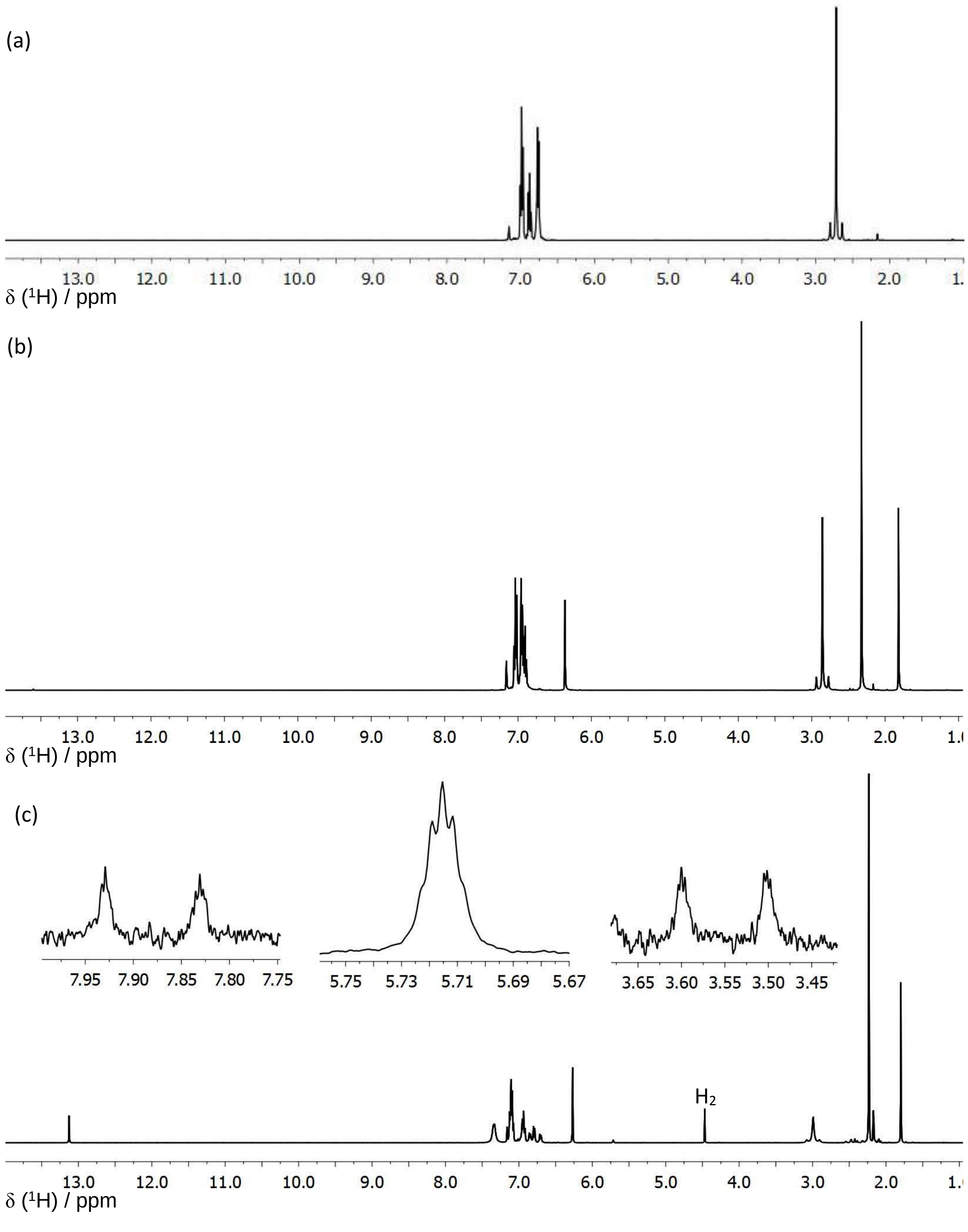

Supplementary Figure 5a: ^{1}H NMR spectra of (a) **1**, (b) **1** and collidine and (c) after admission and activation of H_2 (4 bar) in C_6D_6. Insets show $Bn_3Sn\mathit{H}$ resonance and $^1J(^{117/119}Sn-^1H)$ satellites (generated *in situ*).

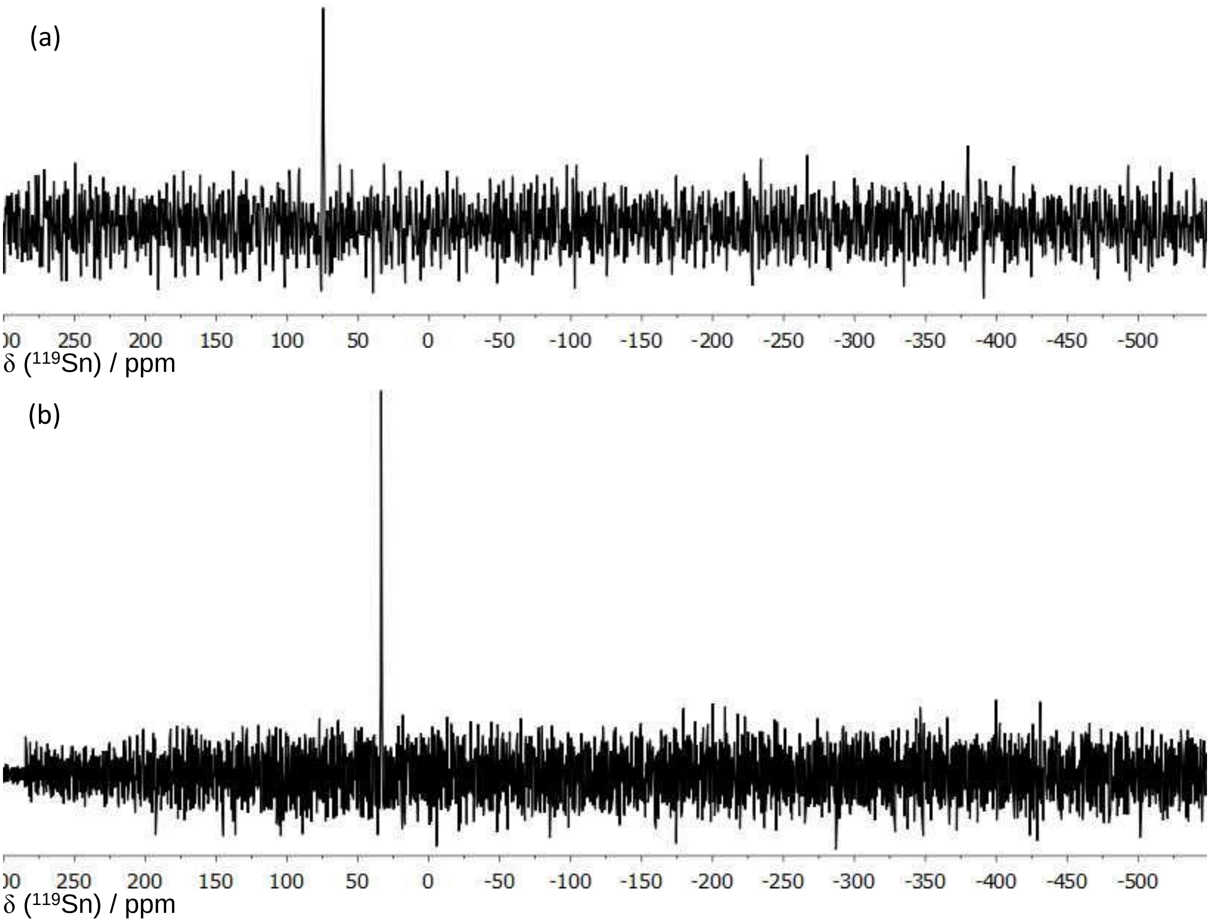

Supplementary Figure 5b: $^{119}Sn\{^1H\}$ NMR spectra of (a) **1** and (b) **1** and collidine in C_6D_6.

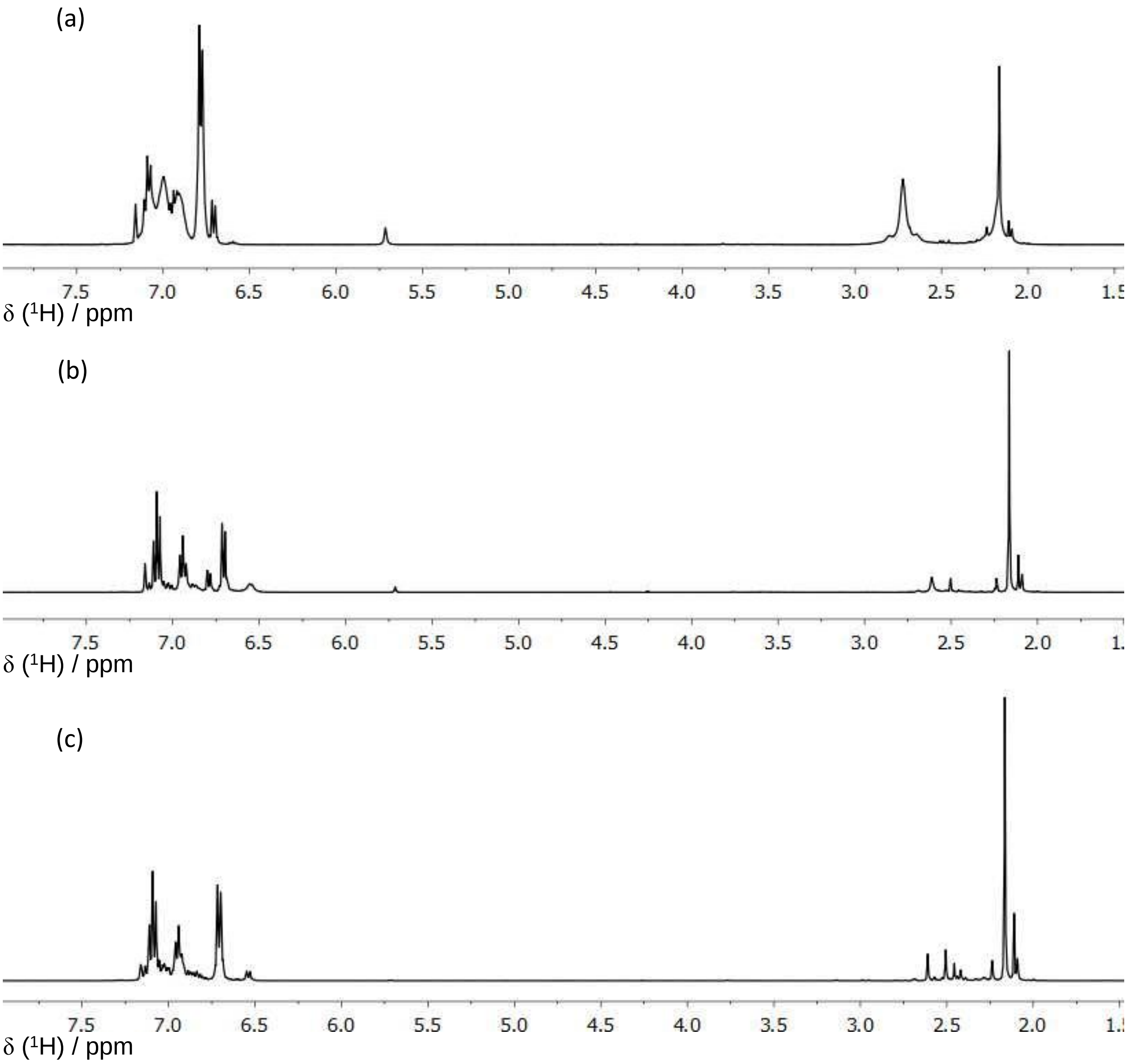

Supplementary Figure 6a: ^{1}H NMR spectra of (a) **1** and **2**, (b) after 60 hours at RT and (c) after 5 hours at 50 ºC.

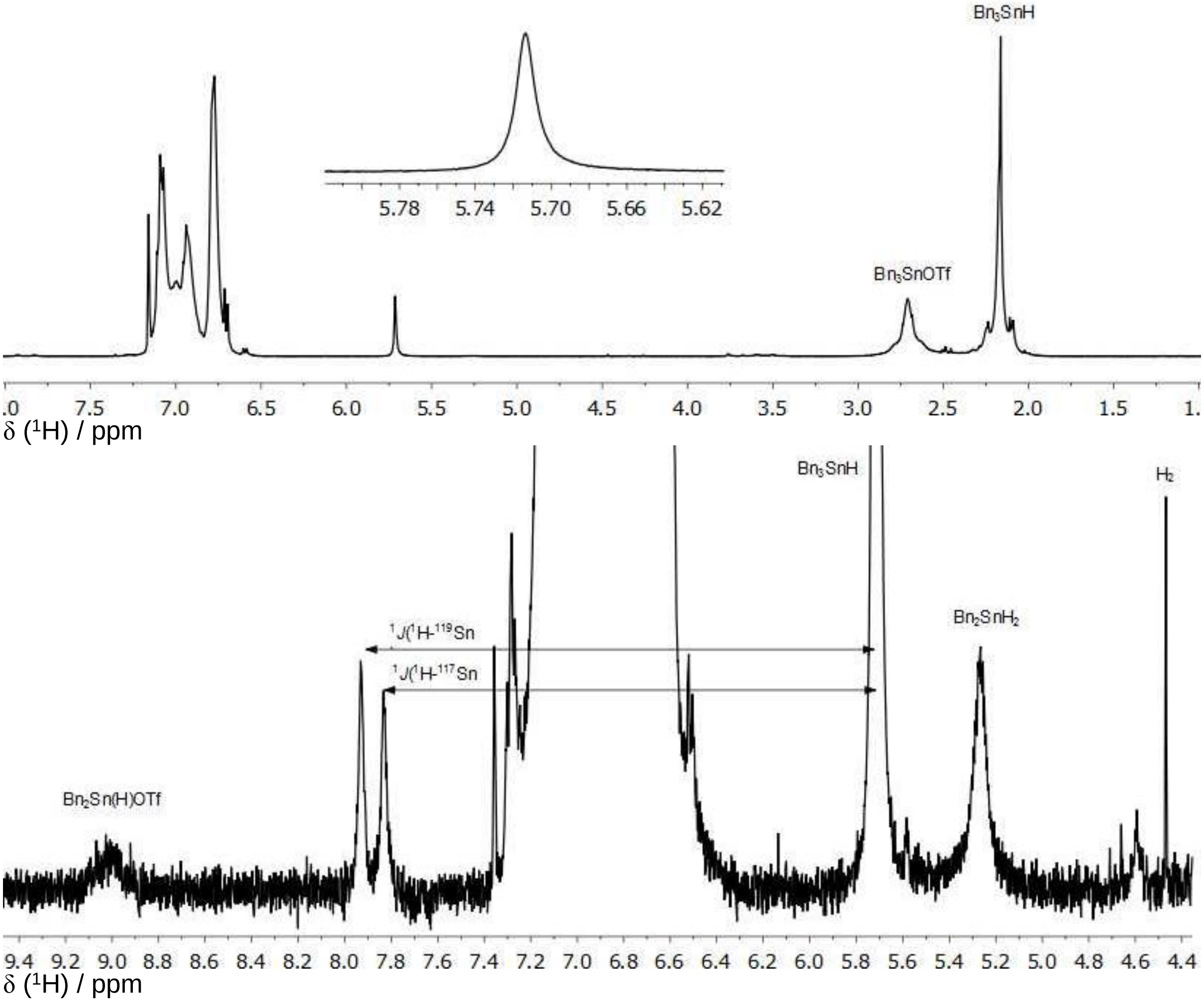

Supplementary Figure 6b: expanded ^{1}H NMR spectra of (a) **1** and **2** [inset shows broad Bn_3Sn*H* resonance] and (b) after 90 min at RT.

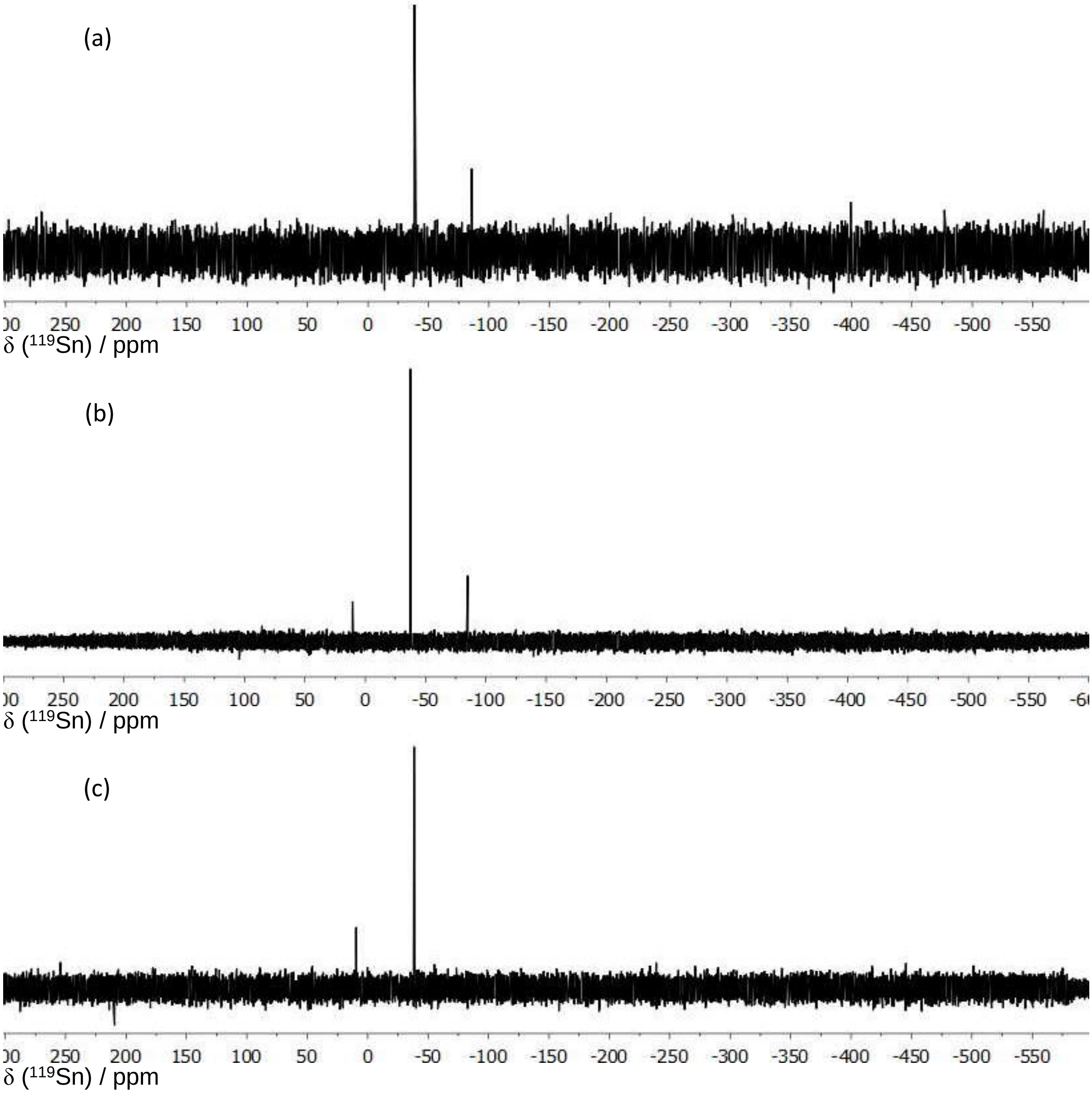

Supplementary Figure 6c: ^{119}Sn{^{1}H} NMR spectra of (a) **1** and **2**, (b) after 60 hours at RT and (c) after 5 hours at 50 ºC (c) in C_6D_6. Peak at δ = +9.5 ppm is due to the presence of $(Bn_3Sn)_2O$ formed from adventitious water (reported δ = +9.2 ppm in C_6D_6 [1]).

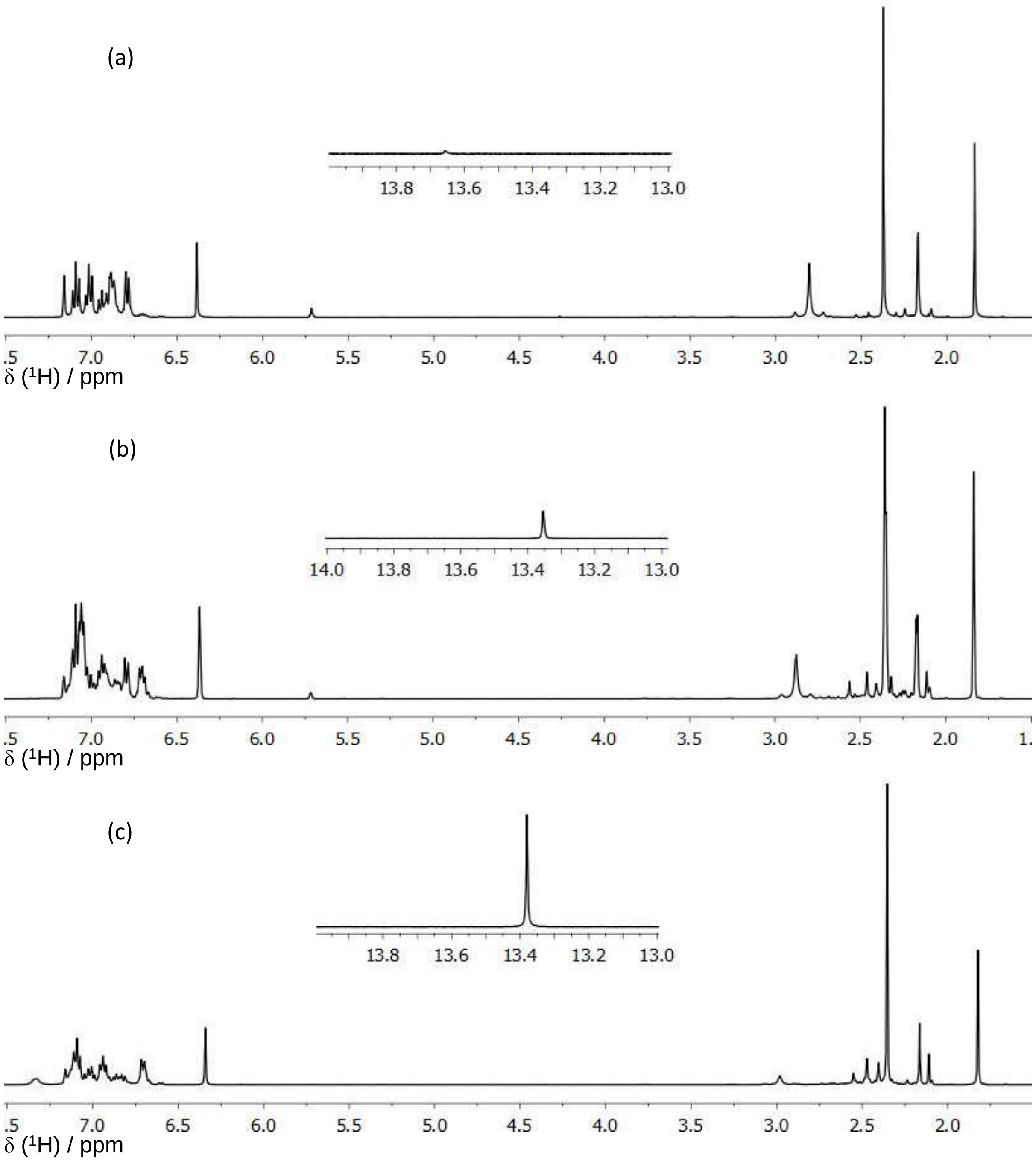

Supplementary Figure 7a: ^{1}H NMR spectra of (a) **1**, **2** and 2 equivalents of collidine, (b) after 60 hours at RT and (c) after 5 hours at 50 ºC. Insets show the growth of the [collidine*H*$^{+}$] resonance as decomposition progresses.

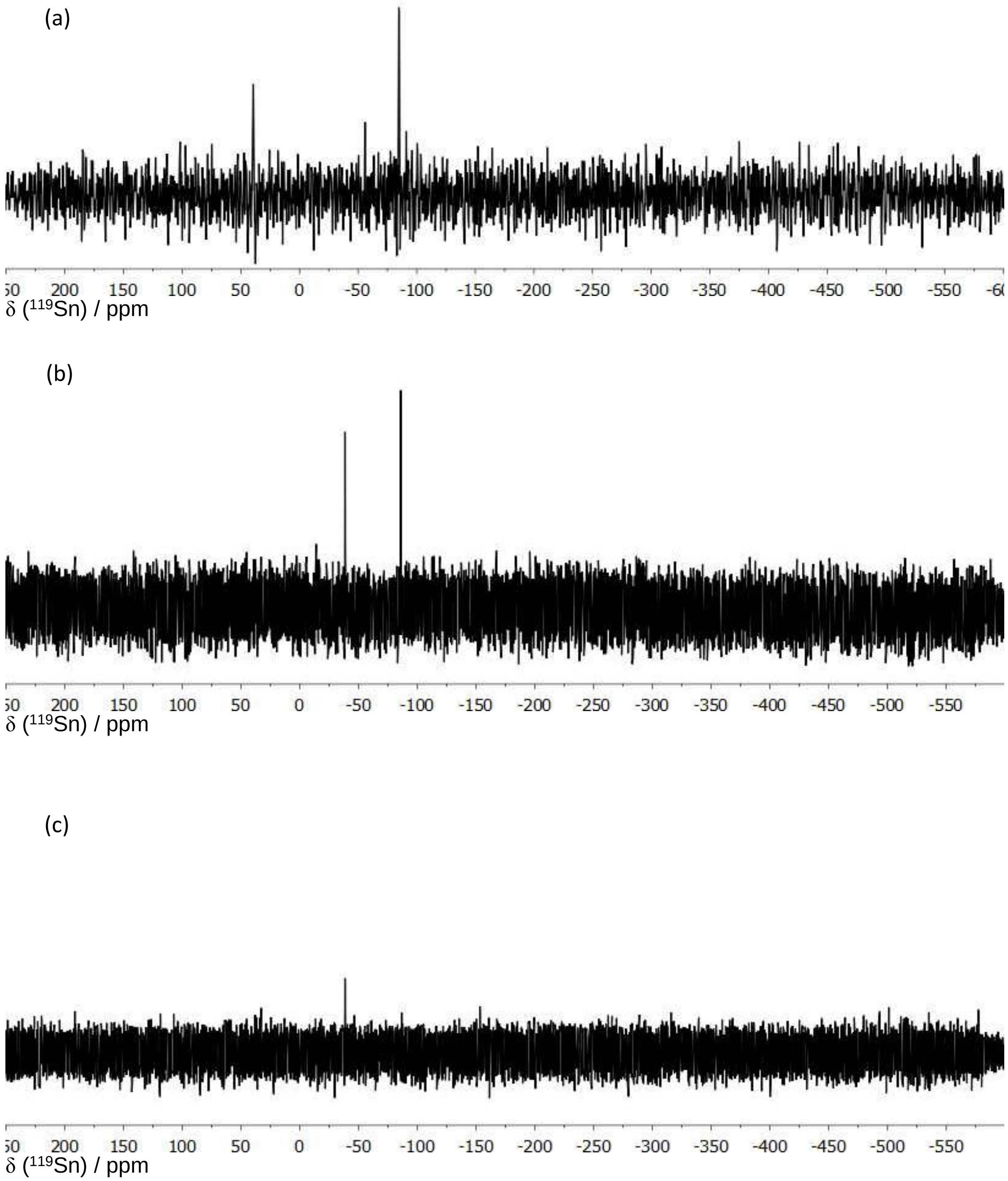

Supplementary Figure 7b: ^{119}Sn{^{1}H} NMR spectra of (a) **1**, **2** and 2 equivalents of collidine, (b) after 60 hours at RT and (c) after 5 hours at 50 ºC (c).

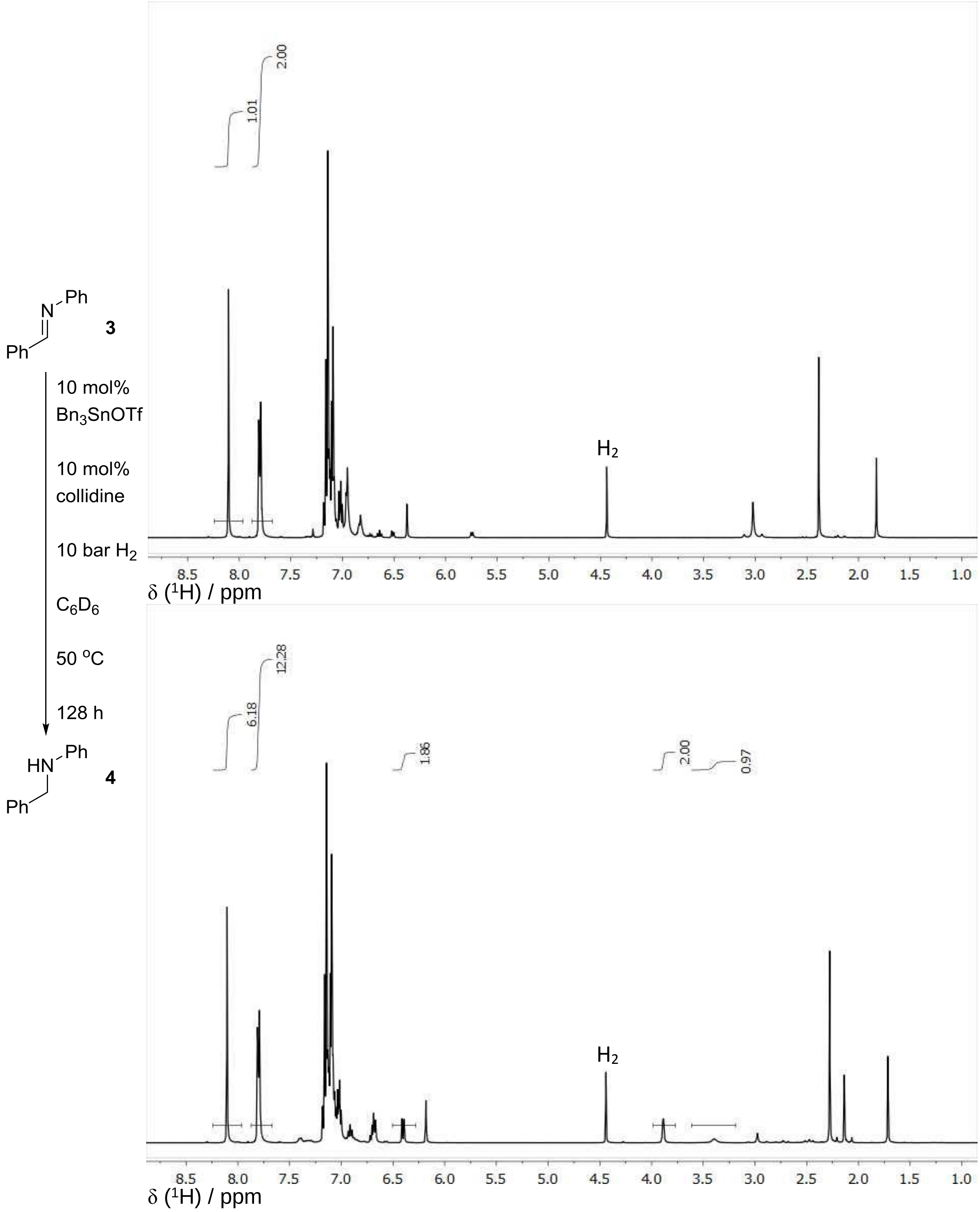

Supplementary Figure 9a: ^{1}H NMR spectra for the hydrogenation of **3** in C_6D_6.

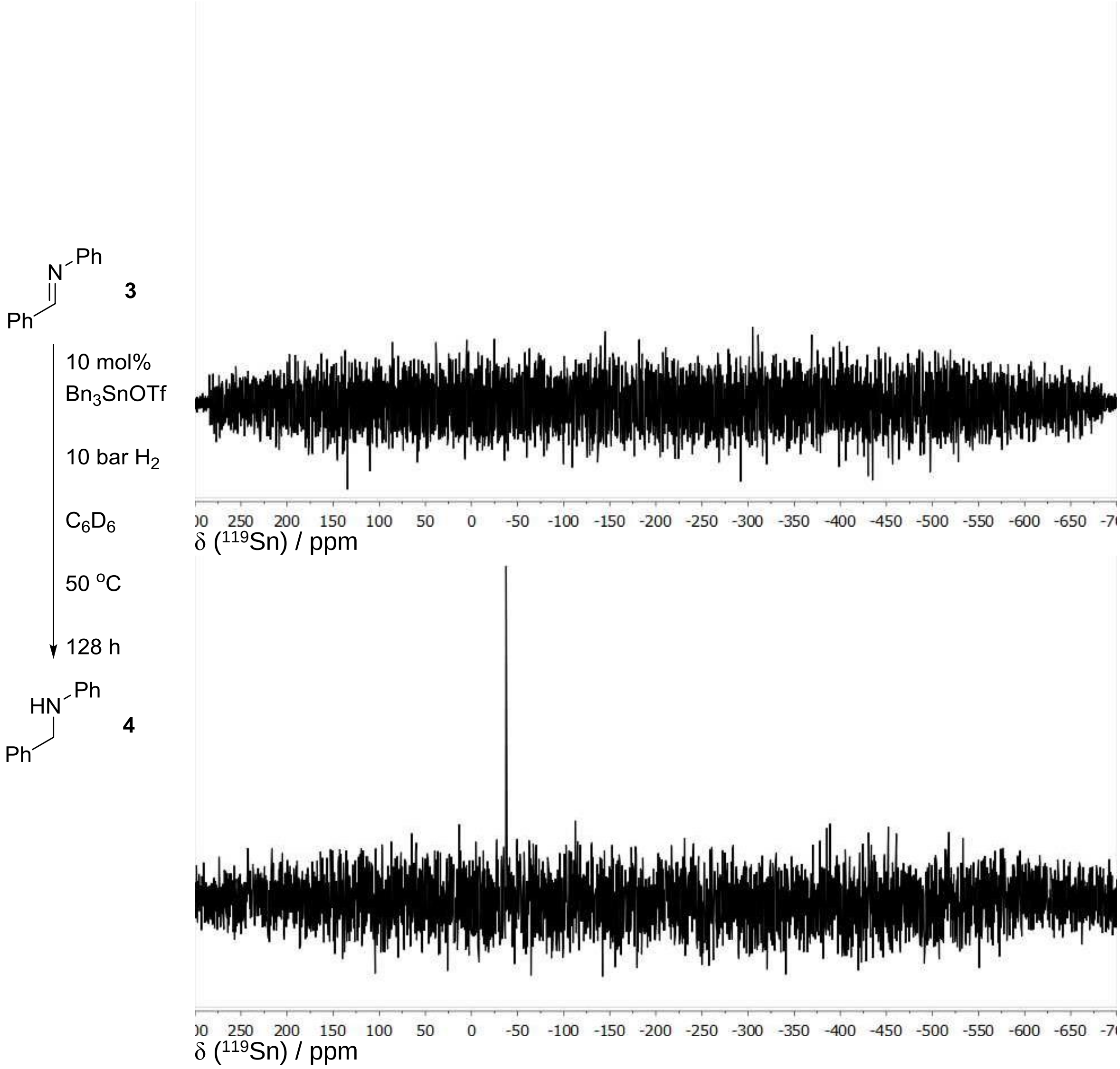

Supplementary Figure 9b: $^{119}Sn\{^1H\}$ NMR spectra for the hydrogenation of **3** in C_6D_6.

The X-ray crystal structure of 1

Crystal data for **1**: $C_{22}H_{21}F_3O_3SSn$, *M* = 541.14, monoclinic, $P2_1$ (no. 4), *a* = 19.5621(8), *b* = 13.1989(3), *c* = 20.0023(8) Å, β = 119.081(5)°, *V* = 4513.5(3) Å^3, *Z* = 8 [4 independent molecules], D_c = 1.593 g cm^{-3}, μ(Mo-Kα) = 1.269 mm^{-1}, *T* = 173 K, colourless blocks, Agilent Xcalibur 3 E diffractometer; 13157 independent measured reflections (R_{int} = 0.0257), F^2 refinement,[2,3] R_1(obs) = 0.0295, wR_2(all) = 0.0617, 12005 independent observed absorption-corrected reflections [$|F_o| > 4\sigma(|F_o|)$, $2\theta_{max}$ = 56°], 1082 parameters. The absolute structure of **1** was determined by use of the Flack parameter [*x* = –0.039(12)]. CCDC 1534626.

The crystal of **1** that was studied was found to be a two component twin in a *ca*. 81:19 ratio, with the two lattices related by the twin law [–1.00 0.00 0.00 0.00 –1.00 0.00 0.99 0.00 1.00], and this was modelled at the refinement stage. The structure contains four independent "molecules" (**1-A**, **1-B**, **1-C** and **1-D**) each of which forms an extended polymer along the crystallographic *b* axis direction.

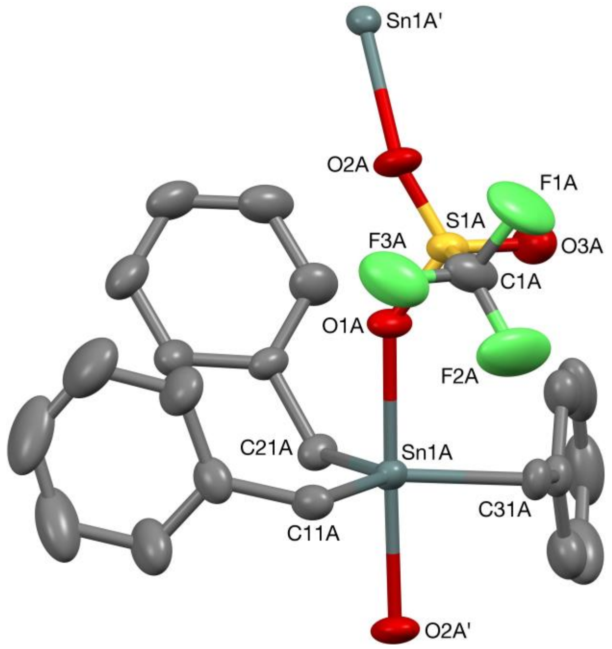

Supplementary Figure 10a: The structure of one (**1-A**) of the four independent "molecules" present in the crystal of **1** (50% probability ellipsoids). The atoms labelled with a prime at the end are related to those without the prime by action of the 2_1 screw axis.

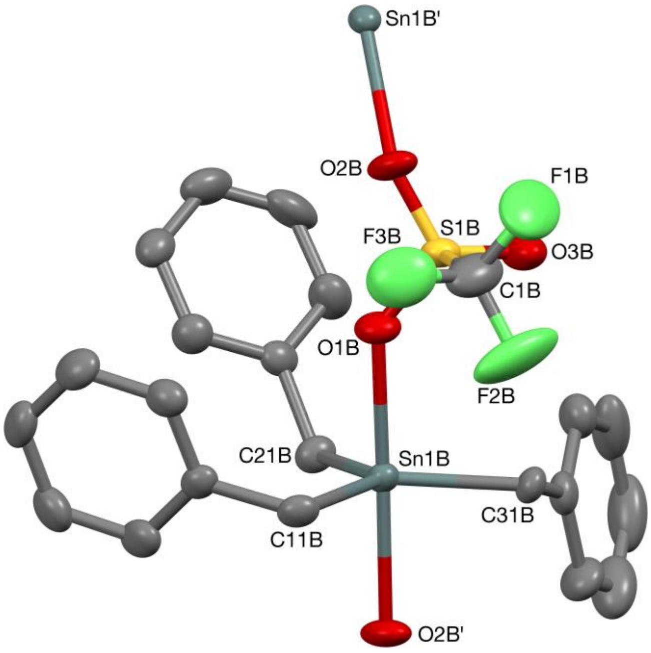

Supplementary Figure 10b: The structure of one (**1-B**) of the four independent “molecules” present in the crystal of **1** (50% probability ellipsoids). The atoms labelled with a prime at the end are related to those without the prime by action of the 2_1 screw axis.

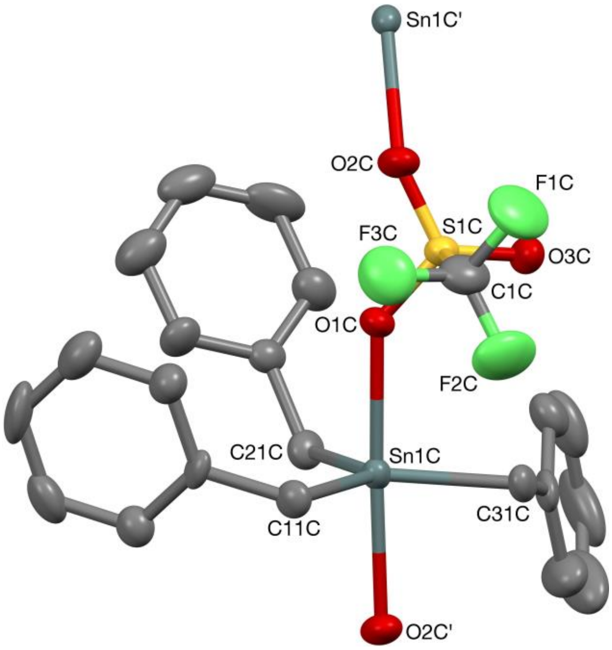

Supplementary Figure 10c: The structure of one (**1-C**) of the four independent “molecules” present in the crystal of **1** (50% probability ellipsoids). The atoms labelled with a prime at the end are related to those without the prime by action of the 2_1 screw axis.

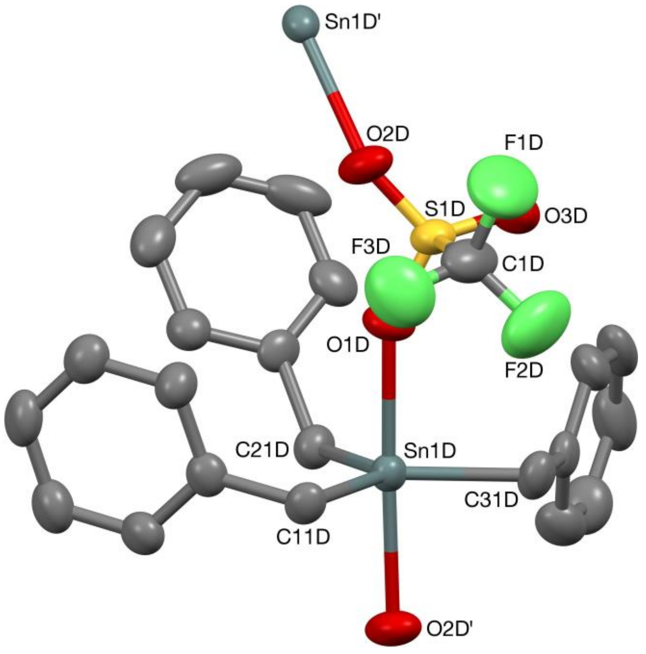

Supplementary Figure 10d: The structure of one (**1-D**) of the four independent “molecules” present in the crystal of **1** (50% probability ellipsoids). The atoms labelled with a prime at the end are related to those without the prime by action of the 2_1 screw axis.

References

[1] A. Lyčka, J. Jirman and A. Koloničný. 1987. *J. Organomet. Chem.* **333**, 305-315. (doi: 10.1016/S0022-328X(00)99807-3)

[2] SHELXTL v5.1, Bruker AXS, Madison, WI, 1998.

[3] SHELX-2013, G.M. Sheldrick, *Acta Cryst.*, 2015, **C71**, 3-8. (doi: 10.1107/S2053229614024218)

© The Authors under the terms of the Creative Commons Attribution License http://creativecommons.org/licenses/by/3.0/, which permits unrestricted use, provided the original author and source are credited.
